# A pH-dependent Antibacterial Peptide Release Nano-system Blocks Tumor Growth *in vivo* without Toxicity

**DOI:** 10.1038/s41598-017-11687-y

**Published:** 2017-09-11

**Authors:** Jing Cao, Yan Zhang, Yanke Shan, Jingui Wang, Fei Liu, Hongrui Liu, Gang Xing, Jing Lei, Jiyong Zhou

**Affiliations:** 1 Engineering Laboratory of Animal Immunity of Jiangsu Province, Institute of Immunology and College of Veterinary Medicine, Nanjing Agricultural University, Nanjing, Jiangsu 210095 P.R. China; 2 College of Biology and Food Science, Shangqiu Normal University, Shangqiu, Henan 476000 P.R. China; 3 Shandong Provincial Key Laboratory of Fine Chemicals, School of Chemistry and Pharmaceutical Engineering, Qilu University of Technology, Jinan, P.R. China

## Abstract

In this study, we designed a nano-system where a novel antibacterial peptide RGD-hylin a1 with reduced hemolysis than the commonly studied melittin was loaded onto mesoporous silica (HMS). We found out that the designed nano-system, RGD-hylin a1-HMS, released RGD-hylin a1 in a pH-dependent manner. It caused apoptosis of cancer cells at low dosage of the antibacterial peptide at pH = 5.5, but was safe to the cells at pH = 7. The hemolytic activity of RGD-hylin a1 itself was reduced by 50~100% by the nano-system depending on the dosage. When this nano-system was administered to tumor-bearing mice at low dosage via intravenous injection, the growth of the solid tumor was blocked by the RGD-hylin a1-HMS nano-system with a 50–60% inhibition rate relative to the PBS-treated control group in terms of tumor volume and weight. Further, the hemolytic activity of RGD-hylin a1 was completely eliminated within the delivery system with no other side effects observed. This study demonstrates that this smart pH-dependent antibacterial peptide release nano-system has superior potential for solid tumor treatments through intravenous administration. This smart-releasing system has great potential in further clinical applications.

## Introduction

Antibacterial peptides are short peptides with a broad spectrum of antibacterial, antifungal and antiviral activities^1^. Some antibacterial peptides are considered ideal antitumor agents because of their anti-resistance ability^2^. For instance, Cecropin A and B, Magainin, melittin, Tachyplesin I and PR-39 of the cathelicidin family have been shown to have favorable antitumor activity^3, 4^. However, their application is limited to hemolysis *in vivo* ^5, 6^. Sequences of antibacterial peptides are modified to reduce hemolytic activity. Yet after modification, the antitumor activities of antibacterial peptides either decrease with hemolytic activities, or become strengthened at the cost of stronger hemolytic activities, thus modification of the sequences alone can hardly lead to a desirable result^7–10^.

Nanomaterials have been widely used for targeted antitumor therapies^11–14^. Compared with normal tissues, tumor tissues have high permeability and retention effect, which contributes to the clustering of nano particles. Many researchers chose nanomaterials as carriers to reduce the hemolytic activities and enhance the tumor-targeting properties of antibacterial peptides. Examples of nanomaterials include polyethyleneglycol-stablized lipid disks^12^, nano particles of polymer materials^15^, quantum dots^11^ and so on. However, there are several drawbacks associated with these designs. An example is for quantum dots, the agents are loaded onto the surface of the carrier particles thus it is difficult to reduce toxicities^16^. One the other hand, liposomes, which can envelop drugs, is susceptible to degradation during *in vivo* transport, thus cannot deliver all the drug carried to the target site^17^. Besides, another major problem is that some carriers’ loading capacities are lower than the effective concentrations (EC) of the carried drugs^18, 19^. Mesoporous materials have highly-ordered pores, high porosity and specific area. Drugs in the pores can be targeted to the tumor tissue and released smartly via external stimulus such as change in pH, where in tumors(pH~6.5)^20^ and endosomes and lysosomes (pH~5–6)^21^. It’s easier for the drugs mesoporous materials carried to reach EC thus reducing the adverse reactions of the drugs^22^. Thus it is intriguing to investigate whether mesoporous materials are suitable carriers for antibacterial peptides in *in vivo* applications.

However, the limitation of the pore size of mesoporous materials causes large antibacterial peptides hard to be loaded. Hence, mesoporous materials are suitable as carriers for antibacterial peptides with high anti-tumor activities and small molecular weights. In this study Hylin a1, whose sequence is IFGAILPLALGALKNLIK, was chosen^23^. Hylin a1 was first separated from the skin of South American tree frogs in 2009^23^. Its relatively small molecular weight contributes to its loading ability onto the mesoporous silica. It has been shown that Hylin a1 has strong antibacterial effect^23^, but its antitumor effect has not yet been reported. In this study we evaluated the antitumor effect of hylin a1 for the first time. We also conjugated a short peptide, RGD (Arg-Gly-Asp), which has high binding activity with multiple tumor cells^24, 25^, with hylin a1 as a tumor targeting head (RGD-hylin a1). Because of the small molecular weight, low immunoreactivity and high specificity of RGD, the antitumor effect of the peptide it’s linked to is enhanced *in vivo* ^13, 14, 26, 27^. According to the molecular weight of RGD-hylin a1, mesoporous silica was synthesized with 150 nm particle size, 23.1 nm pore size, and a –COOH (HMS-COOH) functional group^28^.

This study demonstrated for the first time that RGD-hylin a1 loaded onto mesoporous silica (RGD-hylin a1-HMS) can inhibit tumor growth efficiently with only few side effects *in vivo*. Initially the study found that both hylin a1 and RGD-hylin a1 had antitumor activity *in vitro*. Next the apoptosis of tumor cells and hemolysis of blood cells caused by RGD-hylin a1 and hylin a1 was compared. RGD-hylin a1 was selected as the loading drug for mesoporous silica. Further, RGD-hylin a1 was enriched in the pores of HMS-COOH via electrostatic interactions. Comparison of the effects of RGD-hylin-a1-HMS and RGD-hylin a1 on tumor cell apoptosis and hemolysis in different pH *in vitro* was done, and evaluation of their antitumor activity *in vivo* investigated. The results indicated that RGD-hylin-a1-HMS realized pH-dependent drug release and reduced the hemolysis of RGD-hylin a1 both *in vitro* and *in vivo*. It also inhibited tumor growth remarkably with no obvious hemolytic activity and lesion on normal cells *in vivo*.

## Results

### Hylin a1 and RGD-hylin a1 induced apoptosis in Hela and Hep2 cells

**Figure 1:**
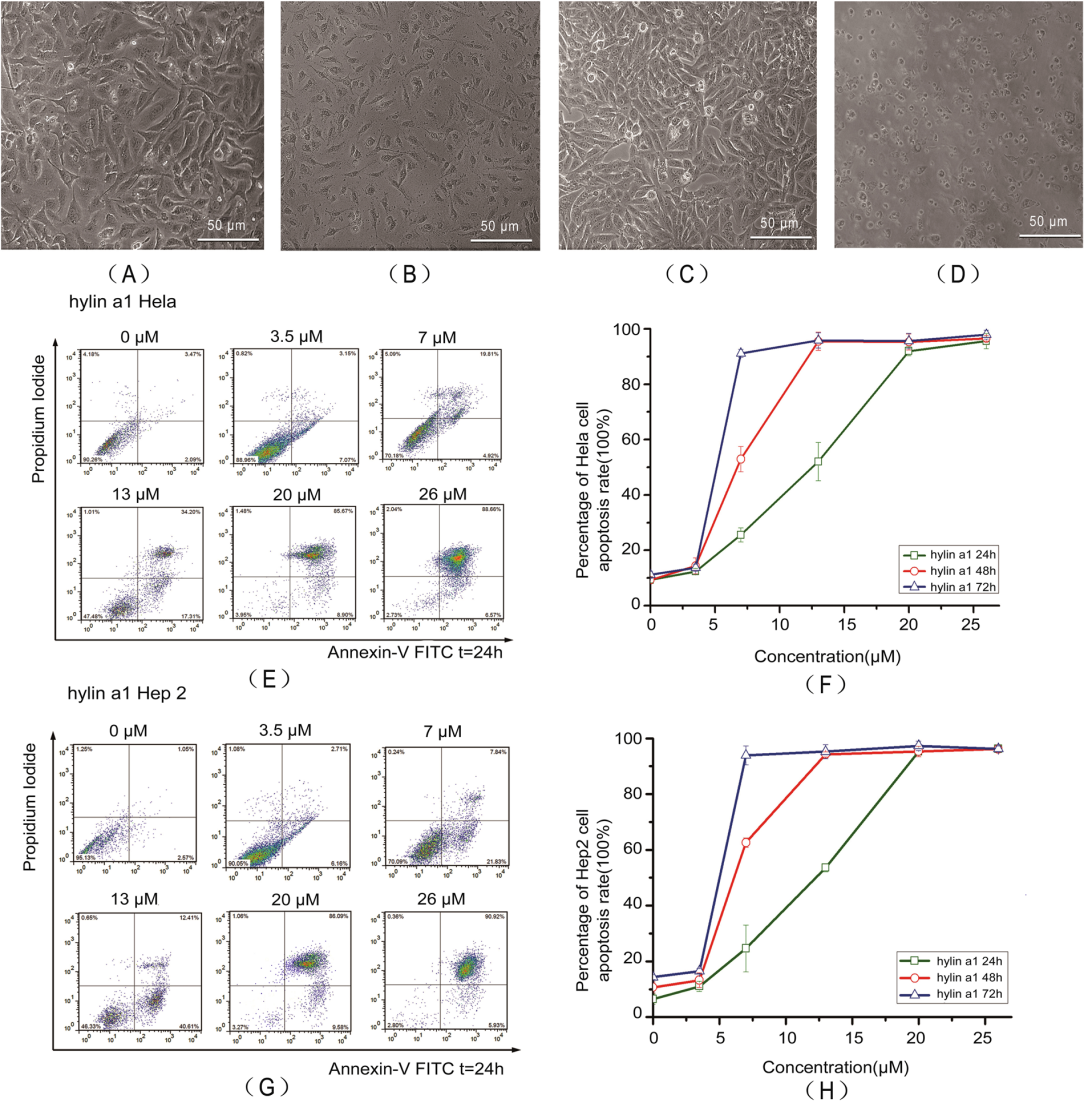
Hylin a1 cytotoxicity assays in Hela and Hep2 cells *in vitro*. Cellular morphology was observed by 10 × inverted microscope. All data was presented as the means ± SD, n = 3. (**A**) Hela cells grew for 24 h. (**B**) Hela cells were treated with 20 μM hylin a1 for 24 h. (**C**) Hep2 cells grew for 24 h. (**D**) Hep2 cells were treated with 20 μM hylin a1 for 24 h. (**E**) and (**F**) Apoptosis assays evaluating the cytotoxicity of hylin a1 in Hela cells. (**G**) and (**H**) Apoptosis assays evaluating the cytotoxicity of hylin a1 in Hep2 cells.

Cell viability in Hela and Hep2 cells as measured by trypan blue staining and cell counting was more than 92%. The Hela and Hep2 cells showed karyopyknosis and apoptotic bodies after treatment with 20 μM hylin a1 for 24 h (Fig. 1(B) and (D)). The results indicated that hylin a1 treatment induced apoptosis in Hela and Hep2 cells. Furthermore, as shown in Fig. 1(E–G), the apoptosis rates of Hela cells and Hep2 cells increased significantly with time (p < 0.05) with increasing concentrations of hylin a1 (p < 0.05). The cells apoptosis reached almost 100% with 20 μM hylin a1 treatment for 24 h. From our results, hylin a1 had strong cytotoxicity to both Hela and Hep2 cells.

**Figure 2:**
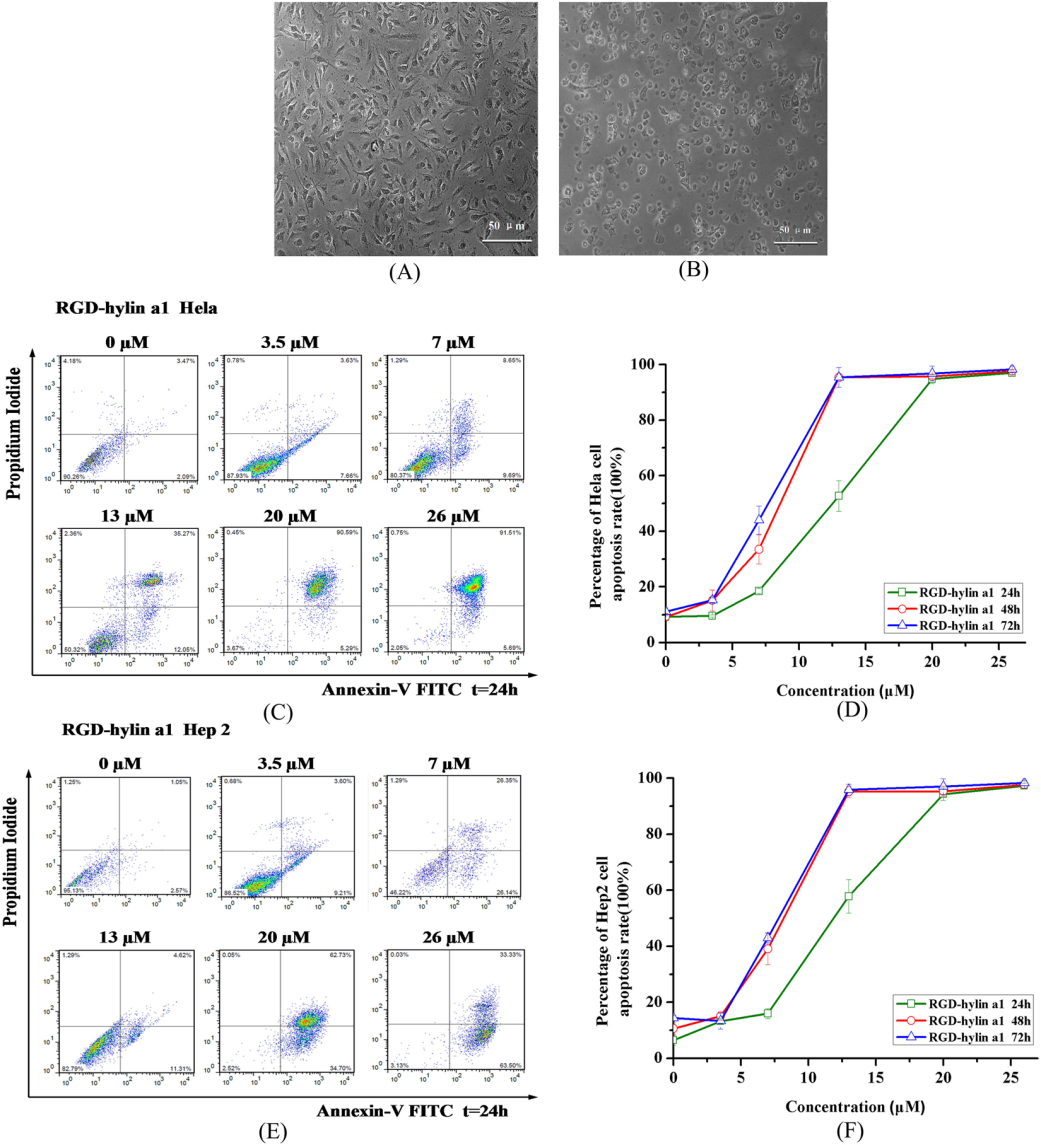
RGD-hylin a1 cytotoxicity assays in Hela and Hep2 cells *in vitro*. Cellular morphology was observed by 10× inverted microscope. All data was presented as the means ± SD, n = 3. (**A**) Hela cells were treated with 20 μM RGD-hylin a1 for 24 h. (**B**) Hep2 cells were treated with 20 μM RGD-hylin a1 for 24 h. (**C**) and (**D**) Apoptosis assays evaluating the cytotoxicity of RGD-hylin a1 in Hela cells. (**E**) and (**F**) Apoptosis assays evaluating the cytotoxicity of RGD-hylin a1 in Hep2 cells.

Similarly, the cytotoxicity of RGD-hylin a1 to Hela and Hep2 cells was also tested. As shown in Fig. 2(A,B), both Hela and Hep2 cells did undergo apoptosis after RGD-hylin a1 treatment, and cell apoptosis rates increased significantly as time and concentration of RGD-hylin a1 increased (Fig. 2(C–F)) (p < 0.05). The cells apoptosis reached around 90% with 20 μM RGD-hylin a1 treatments for 24 h. Thus RGD-hylin a1 also had strong cytotoxicity to Hela and Hep2 cells as hylin a1, meaning that the addition of RGD to hylin a1 did not change the cytotoxicity of the antibacterial peptide to the cells significantly.

Measurement of the mitochondrial membrane potential was done to verify whether apoptosis was induced in the cells before and after peptides treatment. A decrease in the mitochondrial membrane potential is an important feature of apoptosis. We observed that the mitochondrial membrane potential of Hela cells was decreased by both hylin a1 and RGD-hylin a1. As shown in Figure S1(A) and (B), comparing 13 μM and 7 μM with the control group, Hela cells mitochondrial membrane potential decreased by 44.79% and 10.55% after hylin a1 treatment respectively, and decreased by 48.39% and 19.03% respectively after RGD-hylin a1 treatment.

To select a suitable drug for mesoporous silica, we compared cytotoxicity and hemolytic activity of hylin a1 and RGD-hylin a1 to tumor cells. Figure S2(A) and (B), shows hylin a1 and RGD-hylin a1 had no significant differences in 24 h at all concentrations tested and at 1.5, 3.5, 13 and 20 μM in 48 h and 72 h (p > 0.05). However there was a significant difference at 7 μM in 48 h and 72 h (p < 0.05). Hylin a1 had 20% and 45% higher apoptosis rate respectively than RGD-hylin a1. For hemolytic activity, hylin a1 and RGD-hylin a1 had no significant differences at 1.5, 3.5, 13 and 20 μM (p > 0.05), however there was a significant difference at 7 μM. The hemolysis rates were 38.7% and 19.7% (p < 0.05) for hylin a1 and RGD-hylin a1 respectively Figure S2(C).

Based on these results RGD-hylin a1 was selected as the drug of choice for mesoporous silica to carry. This was after considering the hemolytic activity of RGD-hylin a1 which was lower than hylin a1’s at 7 μM, thus potentially safer for *in vivo* application. In addition, although cytotoxicity in Hela and Hep2 cells of hylin a1 and RGD-hylin a1 had no significant difference in 24 h, in theory, RGD-hylin a1 has anti-tumor targeting ability *in vivo*. Mesoporous silica (HMS-COOH) with 23.1 nm pore diameter was chosen as the carrier based on the molecule weight of RGD-hylin a1 (MW: 2394) mesoporous silica (HMS-COOH)^28^.

### Characterization of HMS and RGD-hylin a1-HMS

**Figure 3:**
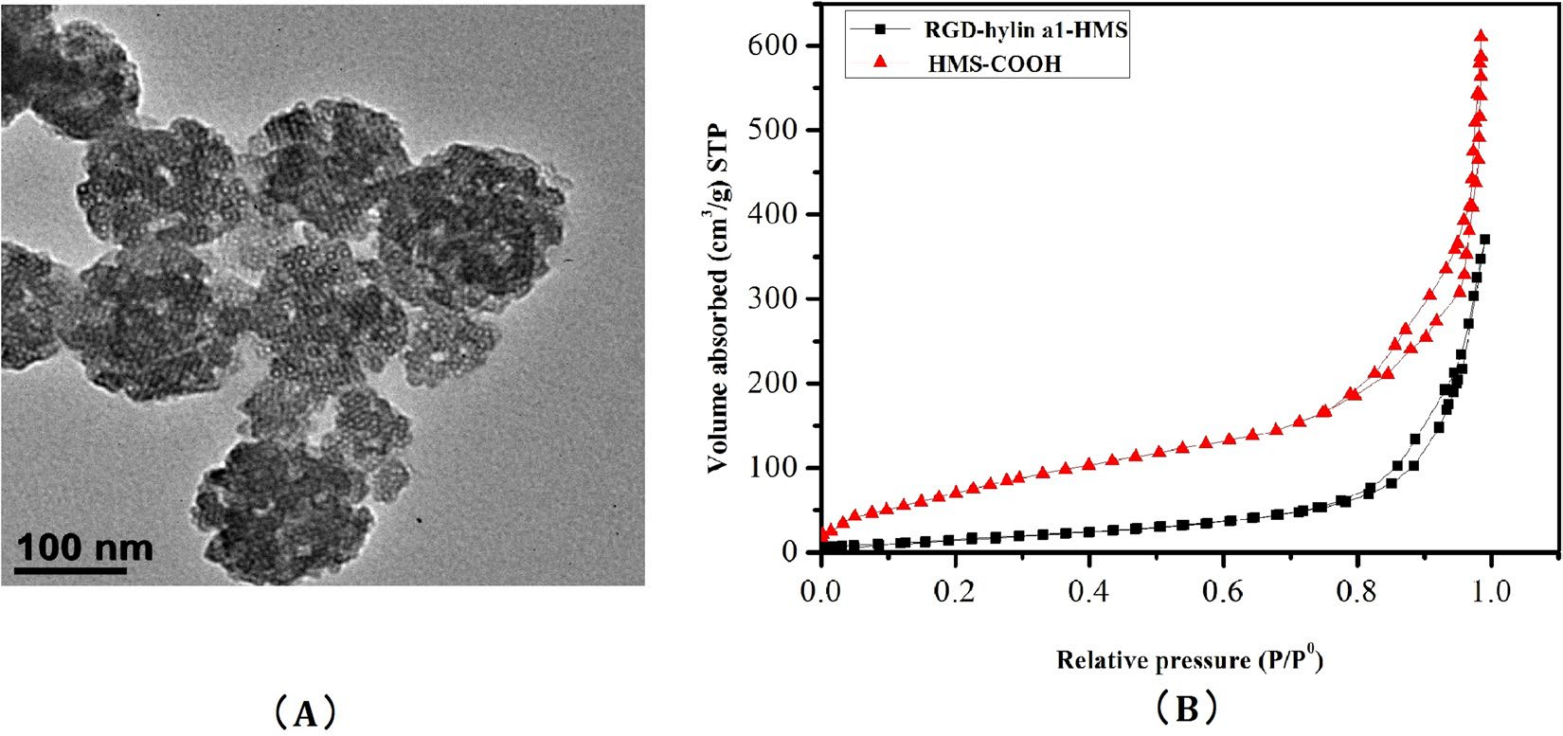
(**A**) Transmission Electron Microscopy (TEM) image of HMS-COOH, the black bar indicates 100 nm. (**B**) Nitrogen adsorption/desorption isotherms of HMS-COOH and RGD-hylina1-HMS samples.

The particle size of mesoporous silica (HMS-COOH) was observed through transmission electron microscopy (TEM) and was about 150 nm (Fig. 3(A)). Nitrogen absorption-desorption experiments provided results for the surface area, desorption volume of the pore, desorption average pore diameter of HMS and RGD-hylin a1-HMS (Fig. 3(B), Table 1). The sample of HMS-COOH exhibited a typical IV isotherm, giving a pore volume of 1.05 cm^3^/g and pore size of 23.1 nm and a specific surface area of 227.4 m^2^/g. RGD-hylin a1-HMS sample still showed a IV isotherm, with a pore volume of 0.18 cm^3^ g^−1^, surface area of 43.6 m^2^ g^−1^, and pore size distribution of 20.4 nm compared to those of HMS-COOH that drastically decreased, which was attributed directly to RGD-hylin a1 stored in the mesopores of the HMS.

**Table 1:** Texatural Parameters obtained from N_2_ adsorption of HMS-COOH and RGD-hylina1-HMS samples.

Sample	BET surface area (m^2.^g^−1^)	BJH desorption volume of pore (cm^3.^g^−1^)	BJH desorption average pore diameter(nm)
HMS-COOH	227.4	1.05	23.1
RGD-hylina1-HMS	43.6	0.18	20.4

### Encapsulation and release rates calculation of RGD-hylin a1-HMS

**Figure 4:**
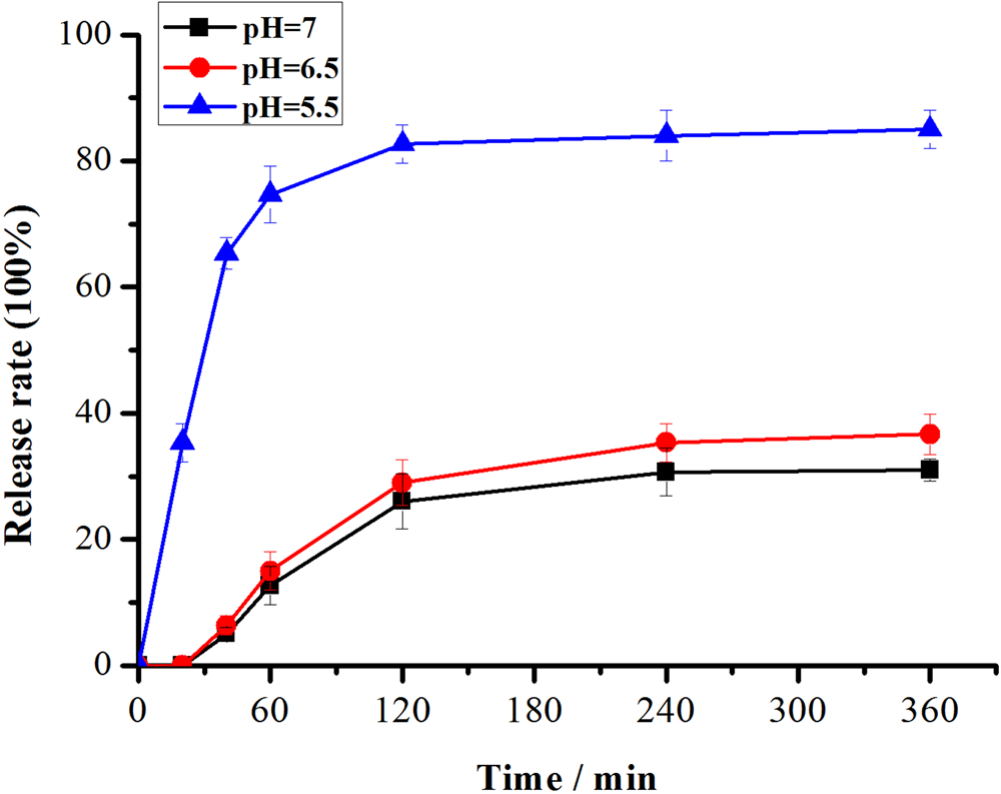
Release rates of RGD-hylina1 from RGD-hylina1-HMS at pH values of 7, 6.5, and 5.5 with different times.

The amount of RGD-hylin a1 for the samples stored inside of HMS was approximately equal to the decreasing amount of RGD-hylin a1 in aqueous solution therefore the released amount of RGD-hylin a1 was approximately equal to the increasing amount of RGD-hylin a1 in aqueous solution. This assisted in measuring the concentration of RGD-hylin a1 by HPLC, followed by analysis of RGD-hylin a1-HMS encapsulation rate and release rates in different times and pHs^29^. We analyzed the release rate of RGD-hylin a1-HMS at pH = 7 (neutral pH), pH = 6.5 (tumor environment) and pH = 5.5 (in endosomes/lysosomes). The results showed that at pH = 7, RGD-hylin a1-HMS had about 31% release ratio in 4 h and 6 h at pH = 7, 35% and 37% release ratios in 4 h and 6 h at pH = 6.5. In comparison, at pH = 5.5, RGD-hylin a1-HMS had 35% release ratio in 20 min, 75% in 1 h, and 84% and 85% in 4 h and 6 h (Fig. 4). From these results we observed a pH-dependent release activity of RGD-hylin a1-HMS indicating release of RGD-hylin a1 from HMS-COOH mainly at pH = 5.5. In summary, we speculated that the encapsulation efficiency of the drug delivery system was 54%, the release ratio was 85% at pH = 5.5 for 6 h from Fig. 4 calculated as ref. 30 and the release of the peptide was mainly intracellular. This was followed by observations of RGD-hylin a1-HMS release conditions at pH 5.5 and 7 in cell and animal experiments.

### RGD-hylin a1-HMS induced pH-dependent apoptosis in Hela and Hep2 cells

**Figure 5:**
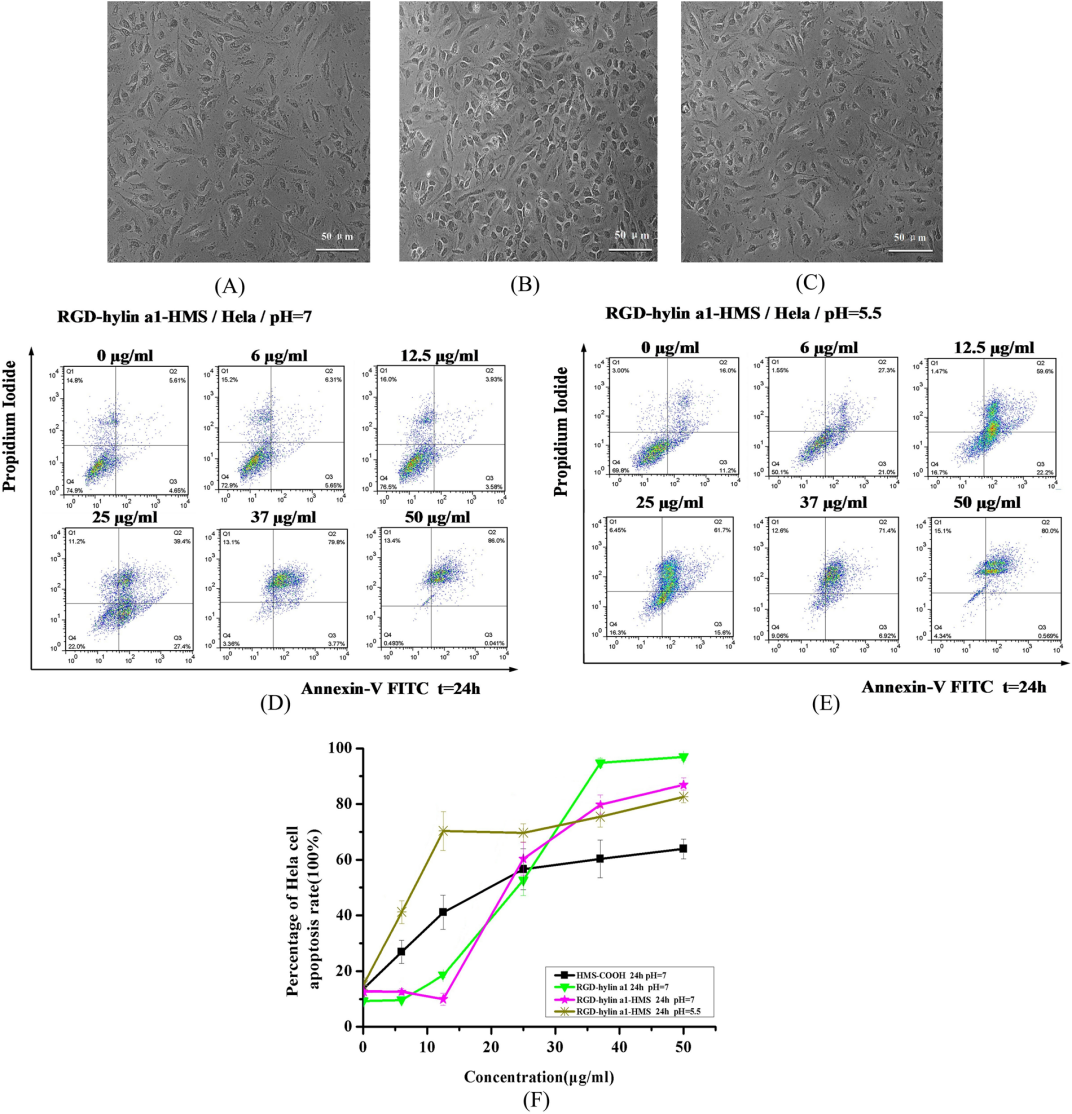
Cytotoxicity assays of HMS-COOH and RGD-hylin a1-HMS (pH = 7 and pH = 5.5) in Hela cells. Cellular morphology was observed by 10× inverted microscope. (**A**) Hela cells were treated with 25 μg/ml HMS-COOH for 24 h; (**B**) Hela cells were treated with 25 μg/ml RGD-hylin a1-HMS (pH = 7) for 24 h; (**C**) Hela cells were treated with 25 μg/ml RGD-hylin a1-HMS (pH = 5.5) for 24 h; (**D**) RGD-hylin a1-HMS induced apoptosis of Hela cells. Hela cells were treated with various concentrations of RGD-hylin a1-HMS (pH = 7) for 24 h. (**E**) RGD-hylin a1-HMS induced apoptosis of Hela cells. Hela cells were treated with various concentrations of RGD-hylin a1-HMS (pH = 5.5) for 24 h. (**F**) Quantification of apoptosis rates of HMS-COOH, RGD-hylin a1, and RGD-hylin a1-HMS (pH = 7 and pH = 5.5) in Hela cells.

The antitumor ability of RGD-hylin a1-HMS at different concentrations and pHs was investigated. As shown in Fig. 5(A–C), 25 μg/ml HMS-COOH, 25 μg/ml RGD-hylin a1-HMS (13 μM, pH = 7) and 25 μg/ml RGD-hylin a1-HMS (13 μM, pH = 5.5) all induced apoptosis in Hela cells. Importantly, HMS-COOH had cytotoxicity in Hela at 6 μg/ml, and the apoptosis rates increased as HMS-COOH concentration increased (Figure S3). It is quite intriguing that at pH = 7 Hela cells didn’t show apoptosis with 6 μg/ml (4 μM) or 12.5 μg/ml (7 μM) RGD-hylin a1-HMS treatment, but at pH = 5.5 the apoptosis rates with 6 μg/ml or 12.5 μg/ml RGD-hylin a1-HMS treatment were 29% or 60% more than that at the same concentration at pH = 7 respectively. The apoptosis rates with 25 μg/ml (12.5 μM), 37 μg/ml (20 μM) or 50 μg/ml (25 μM) RGD-hylin a1-HMS treatment at pH = 5.5 were similar to that at pH = 7 (Fig. 5(D–F)). As shown in Fig. 5(F) Figure S4. The significant cells apoptosis at low concentration and low pH also indicated RGD-hylin a1-HMS had pH dependent release. In addition we observed apoptosis of Hep2 cells at pH = 7 and pH = 5.5 with RGD-hylin a1-HMS (Figure S4).

**Figure 6:**
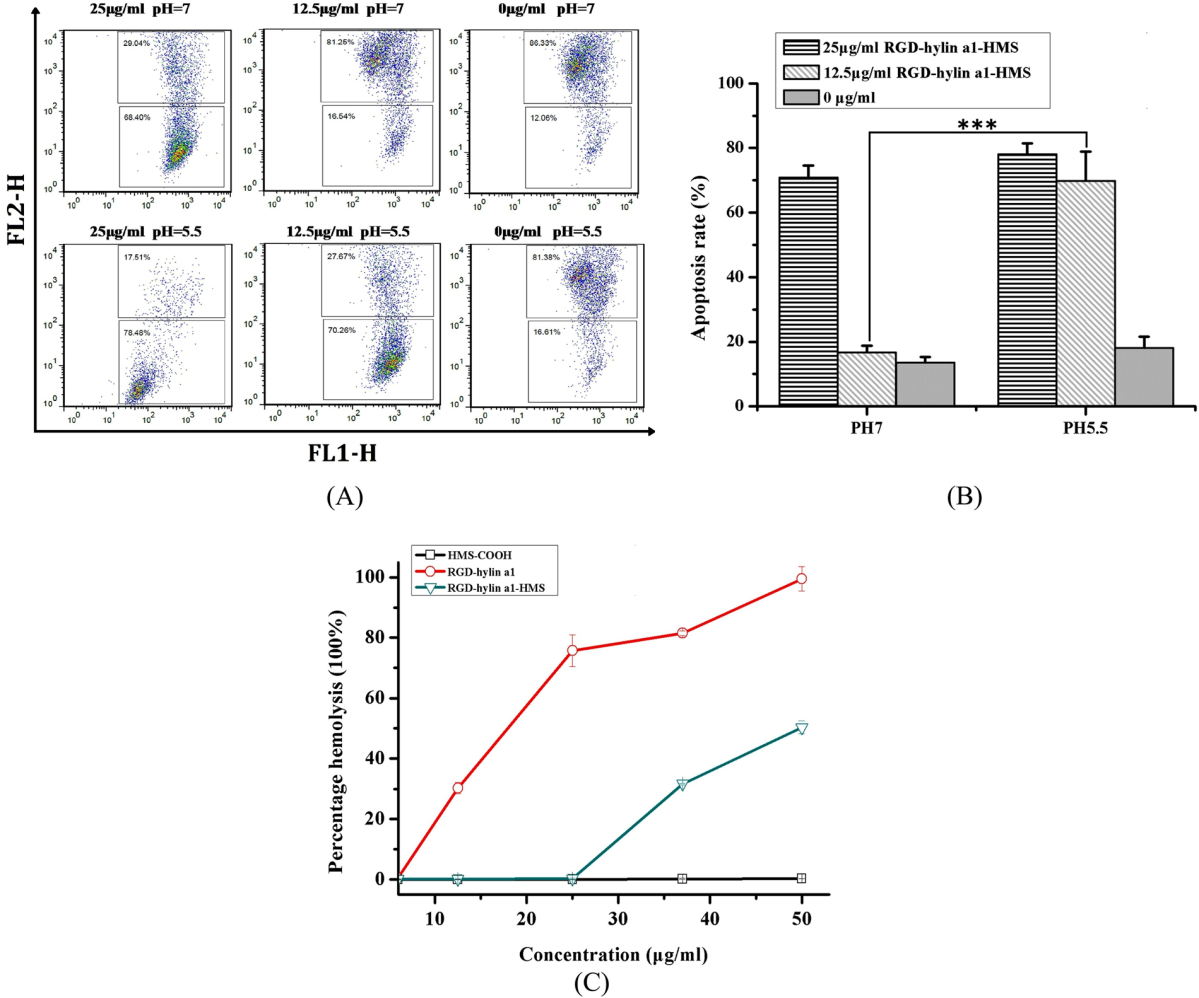
Mitochondrial membrane potential assays and hemolysis assays in Hela cells. All data was presented as the means ± SD, n = 3. (**A**) and (**B**) Hela cells were treated with RGD-hylin a1-HMS for 24 h, stained with JC-1, and analyzed by FACS. Results were presented as percentage of apoptosis. (**C**) Hemolytic assays for HMS-COOH, RGD-hylin a1 and RGD-hylin a1-HMS (pH = 7) in RBC.

To verify the pH dependent release, we measured the mitochondrial membrane potential changes of Hela cells after treatment with RGD-hylin a1-HMS. As shown in Fig. 6(A) and (B), with treatment of 25 μg/ml or 12.5 μg/ml RGD-hylin a1-HMS for 24 h, the mitochondrial membrane potential decreased by 57.22% and 3.13% respectively at pH = 7, and decreased by 59.94% and 51.69% respectively at pH = 5.5, this was consistent with the apoptosis assay that RGD-hylin a1-HMS had a pH dependent release.

Comparison of the hemolytic activity of HMS-COOH, RGD-hylin a1 and RGD-hylin a1-HMS was done. As shown in Fig. 6(C), HMS-COOH did not have any hemolytic activity from 0 μg/ml to 50 μg/ml; the hemolytic ratios induced by RGD-hylin a1 were 30%, 76%, 82% and 99% respectively at 12.5, 25, 37 and 50 μg/ml. RGD-hylin a1-HMS decreased hemolytic activity as no obvious hemolytic activity was observed at 12.5 and 25 μg/ml, and only 32% and 50% hemolytic activity was observed at 37 μg/ml and 50 μg/ml respectively. Based on these results, we concluded that hemolytic activity of RGD-hylin a1 was far much higher than that of RGD-hylin a1-HMS, a possible explanation was due to enriching the antimicrobial peptide RGD-hylin a1 to HMS-COOH contributing to the reduced hemolytic activity.

Considering the apoptosis assay and hemolytic assay we realized that RGD-hylin a1-HMS showed pH-dependent release with no hemolytic activity but with anti-tumor activity at pH = 5.5 and low concentration (~12.5 μg/ml).

### RGD-hylin a1-HMS inhibits tumor growth *in vivo* with low toxicity

**Figure 7:**
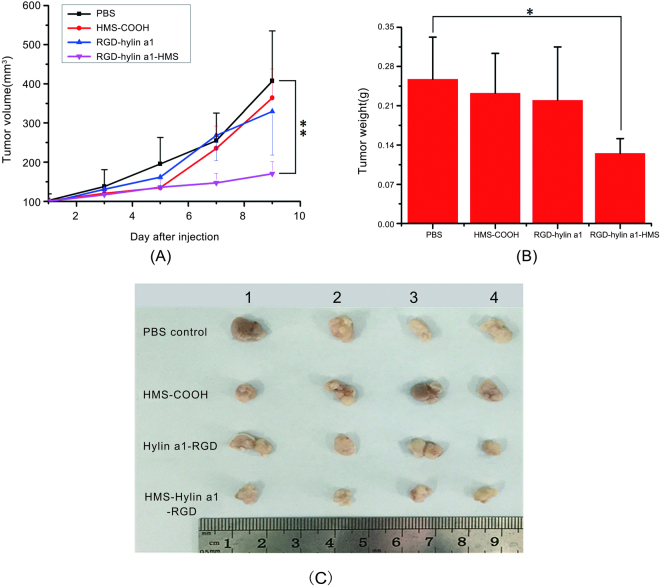
*In vivo* evaluation of the effect of RGD-hylin a1-HMS on the inhibition of tumor growth. All data was presented as the means ± SD, n = 4, **p < 0.01, *p < 0.05. (**A**) and (**B**) Tumor volume and weight in each group with increasing days. (**C**) Photographs of the tumors from each group on the 9th day.

To evaluate the efficacy of RGD-hylin a1-HMS against tumors *in vivo*, Balb/c mice were implanted subcutaneously with 0.2 ml × 10^7^ Hep2 cells in the left flank^31^. As shown in Figs 7 and S5, the mean tumor volume and weight were 50–60% reduced in the RGD-hylin a1-HMS group compared to the PBS control group (p < 0.01, 171 mm^3^ vs 407 mm^3^, n = 4; p < 0.05,0.125 g vs 0.258 g, n = 4) on the 9th day which was 3 days after drug injection was stopped. However HMS-COOH group and RGD-hylin a1 group had no obvious anti-tumor effects (Fig. 7). Moreover, for the RGD-hylin a1 group, the tails of the nude mice festered seriously, while for the other two groups there was no difference with the control group (Figure S5). These results indicated that RGD-hylin a1 had serious hemolytic activity *in vivo*, but loading it onto mesoporous silica (RGD-hylin a1-HMS) decreased its hemolytic activity effectively and making it more bio-safe.

**Figure 8:**
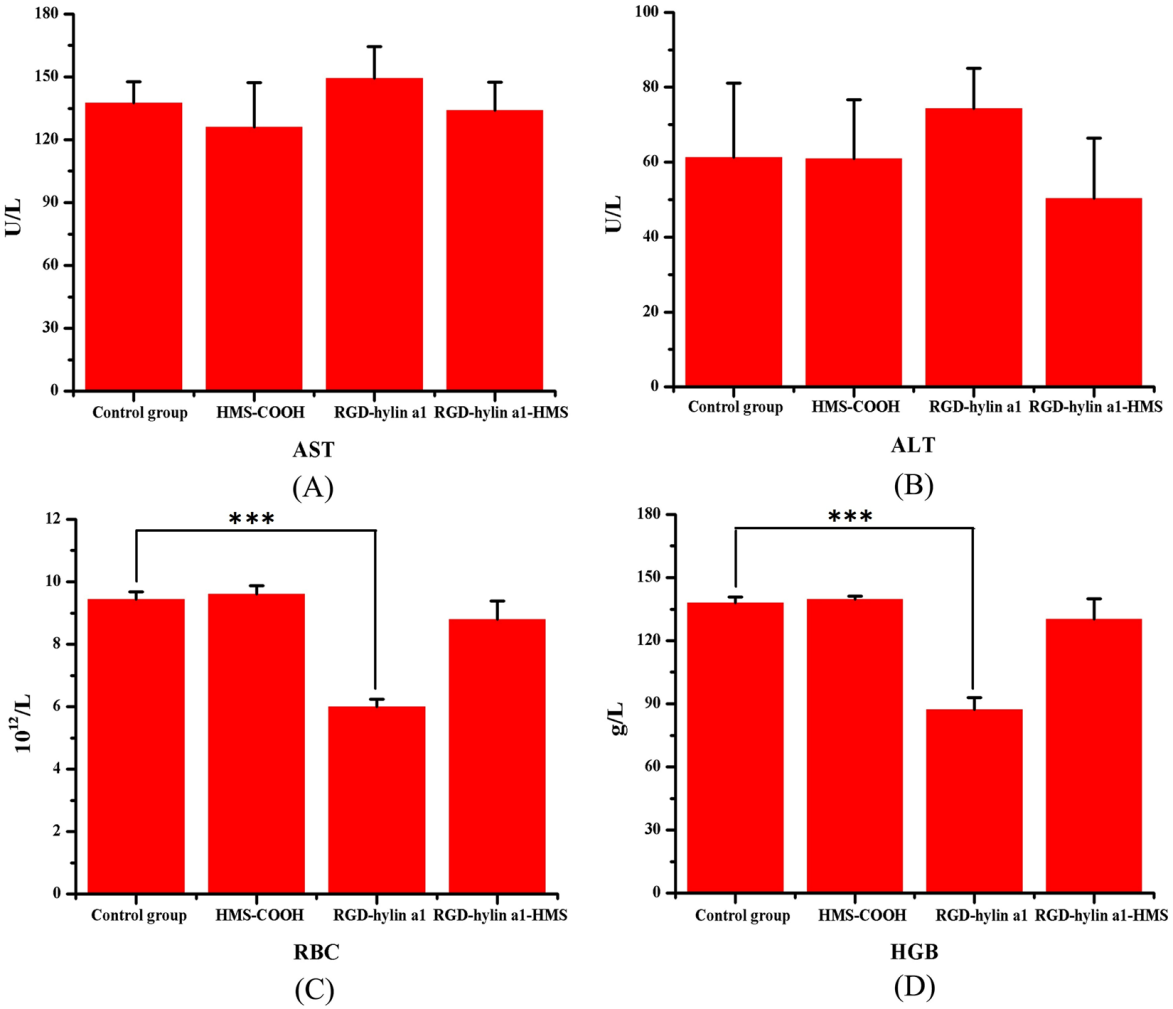
Evaluation of the side effects of RGD-hylin a1-HMS *in vivo*. All data was presented as the means + SD, n = 3, *p < 0.05, **p < 0.01, ***p < 0.001. (**A**,**B**) Biochemical analyses of glutamate pyruvate transaminase (ALT) and glutamic-oxalacetic transaminease (AST). (**C**,**D**) Blood hemanalysis of red blood cells (RBC) and hemoglobin (HGB).

**Figure 9:**
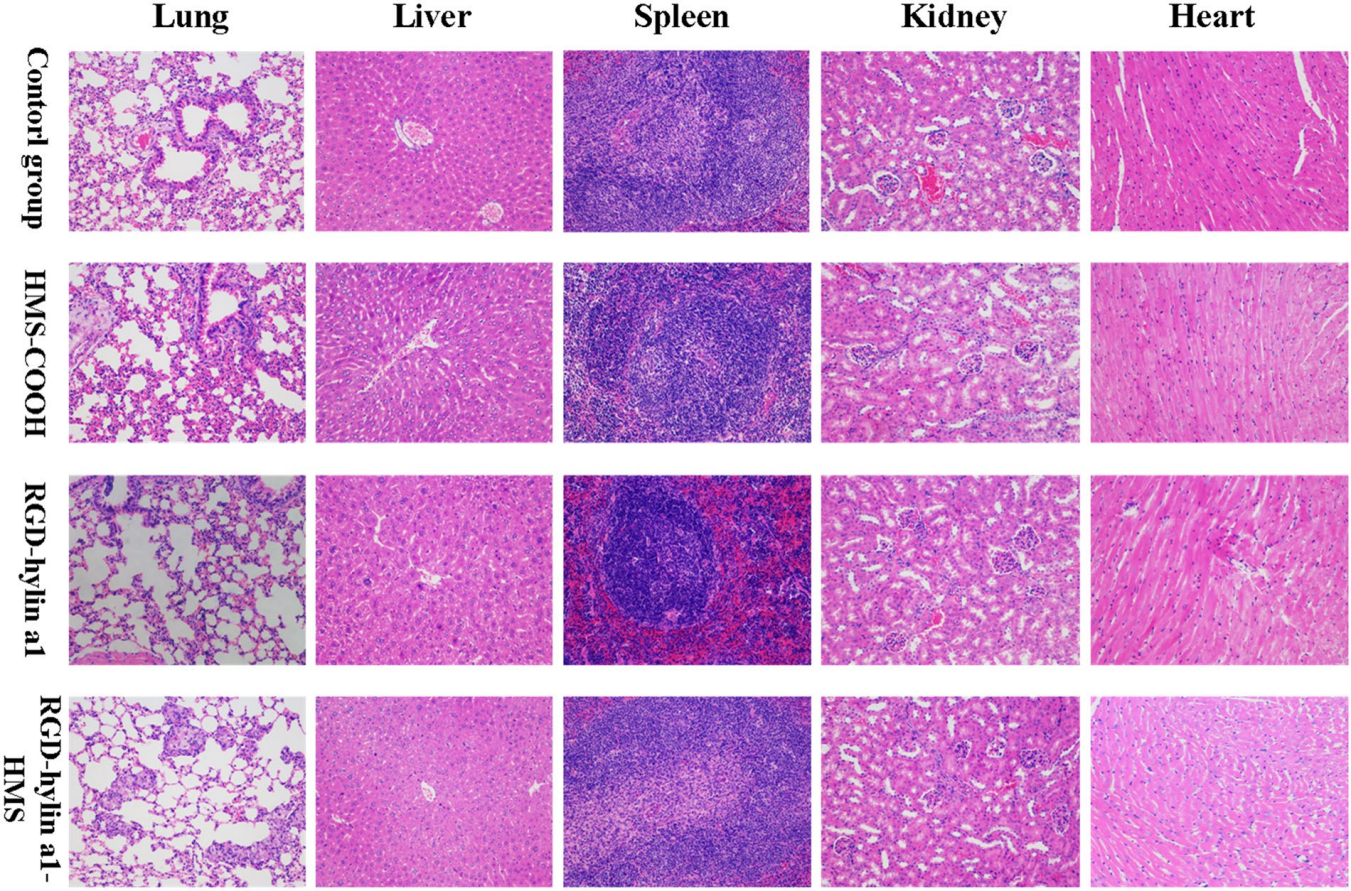
Histopathologic analyses of H&E-stained tissue sections from the hearts, livers, spleens, lungs, and kidneys of tumor-bearing mice.

The biosafety of RGD-hylin a1-HMS was further evaluated by hemanalysis and histopathologic analysis. Blood was collected from the mice on the ninth day for hemanalysis and biochemical analyses, and tissues (hearts, livers, spleens, lungs, and kidneys) were removed for histopathologic analyses. The hemanalysis showed that there were no differences in glutamate pyruvate transaminase (ALT), aspartate aminotransferase (AST), blood urea nitrogen (BUN), white blood cells (WBC), mean cell hemoglobin (MCH), and mean corpuscular hemoglobin concentration (MCHC) among the four groups (p > 0.05) (Fig. 8(A,B), Figure S6), indicating that the three treatment groups had no observable damages to the main organs of the nude mice. However, the red blood cells (RBC) and hemoglobin (HGB) levels in the RGD-hylin a1-treated group were lower than those in PBS-treated control (Fig. 8(C,D)) (p < 0.001). This indicated that RGD-hylin a1-treated group had hemolytic activity *in vivo* which was consistent with the festered tails observed in the nude mice. Images of H&E-stained tissue sections showed that there were no damages in the organs (hearts, liver, spleens, lungs, and kidneys) in the four groups at the given dose (Fig. 9). These results proved that HMS-hylin a1-RGD efficiently inhibited tumor growth and could be used safely *in vivo* without toxicity concerns^31^.

## Discussion

Antimicrobial peptides have a broad spectrum of abilities for resisting bacteria, fungi and some viruses, while some cationic antimicrobial peptides can inhibit tumor growth and enhance the body’s immunity function^3, 4^. In this study we designed a mesoporous silica anti-tumor drugs carrier system with hylin a1 as the core^23^. Our study demonstrated the antitumor activity of hylin a1, an antimicrobial peptide that was previously only been known for its antimicrobial activity. Both apoptosis and mitochondrial membrane potential assays showed that apoptosis was induced in tumor cells with hylin a1 or RGD-hylin a1 treatment. However, the hemolytic activity of RGD-hylin a1 was 20% lower than hylin a1 at 7 μM (p < 0.05) (Figure S2).

To further reduce the hemolytic activity of RGD-hylin a1, mesoporous silica was selected as a drug carrier. Mesoporous silica was generally thought to be biosafe^32, 33^, according to our results (Fig. 5, Figure S3) we found out that HMS-COOH had anti-tumor activity as well. We presumed that HMS-COOH could promote tumor cells apoptosis depending on its functional group (-COOH). The electric polarity of HMS-COOH caused cell apoptosis when HMS-COOH was swallowed by the cells. This was concealed when the amino end of RGD-hylin a1 was combined with -COOH through electrostatic absorption, resulting in a stable neutral charge that makes them avoid interference to proteins^34^, thus more biocompatible^35, 36^. In addition, entrance of RGD-hylin a1 into the holes of HMS-COOH could conceal its toxicity, thus the apoptosis rates of Hela cells and rates obviously decreased by 50~100% with RGD-hylin a1-HMS treatment at lower concentrations and pH = 7. However, the electrostatic absorption was disrupted when cells were in an acidic environment (pH = 5.5), resulting in faster release of RGD-hylin a1 from HMS holes causing higher apoptosis rate and hemolytic activity (Figs 5 and 6). The changes in mitochondrial membrane potential further proved with 12.5 μg/ml RGD-hylin a1-HMS, Hela cells apoptosis rate was significantly higher at pH = 5.5 than that at pH = 7 (Fig. 6).

*In vivo* experiments showed that RGD-hylin a1 group had stronger hemolytic activity to tumor induced nude mice, and the injection site of the tail had severe decay. In comparison, RGD-hylin a1-HMS group had no hemolytic activity to tumor nude mice and the injection site in the tail had no difference from the control group (Fig. 8, Figure S5). These results indicated that RGD-hylin a1-HMS was not excessively released in the blood circulation of the tumor induced nude mice. Further, RGD-hylin a1-HMS had obvious suppression on tumor growth with the inhibitory rate around 50–60%, while in comparison RGD-hylin a1 had no observable suppression on tumor growth (Fig. 7), indicating the 100–150 nm mesoporous silica delivery system could efficiently permeate solid tumors^37^, protect RGD-hylin a1 from degradation in the blood circulation and release RGD-hylin a1 when it reaches the tumor. Hemanalysis and histopathologic analysis showed that RGD-hylin a1-HMS had no influence on several blood biochemical indexes and organs (Fig. 9, Figure S6). In short, the antibacterial peptides RGD-hylin a1 enriched to HMS-COOH were released after RGD-hylin a1-HMS targeted the tumor and achieved a synergistic effect of tumor inhibition with no side effects *in vivo* ^38, 39^.

## Conclusion

In conclusion, a new antibacterial peptide delivery strategy was designed using nanomaterials that could target and inhibit tumor cell growth *in vivo* without causing toxicity. We demonstrated that the cytotoxicity of RGD-hylin a1 was successfully concealed within the delivery system before its release to cancer cells. This smart pH-dependent drug release nano-system, RGD-hylin a1-HMS, has superior potentials for solid tumor treatments and may be applied in delivery of other types of antibacterial peptides through intravenous administration as well.

## Materials and Methods

### Chemicals and Reagents

Hylin a1 and RGD-hylin a1 were synthesized by Ango Biotechnology Co., Ltd. (Shanghai, China). Dimethyl Sulphoxide (DMSO) and Triton-100 were of analytical grade and were used without further purification. These reagents were purchased from Sigma-Aldrich (USA). 0.25% Tripsin was purchased from Wisent (Australia). The fetal bovine serum was purchased from Millipore (USA), and DMEM was purchased from Hyclone (USA). Annexin V FITC Apoptosis Detection Kit (Cat. NO. 556547) and Flow Cytometry Mitochondrial Membrane Potential Detection Kit (Cat. NO. 551302) were purchased from BD (USA). Cetyltrimethylammonium bromide (CTAB) was from Amresco. Poly acrylic acid (PAA, average molecular weight 240,000, 25 wt % solution in water) was from Alfa Aesar. NaCl, Na_2_HPO_4_.2H_2_O, KH_2_PO_4_, and KCl were all of analytical grade. The 10 mM phosphate-buffered saline (PBS) solutions (pH = 7.4) contained 0.01 M sodium phosphate, 0.137 M NaCl, 0.01 M Na_2_HPO_4_.2H_2_O, 0.002 M KH_2_PO_4_ and 0.0027 M KCl. Ultrapure water in the experiment was provided by Milli-Q Academic system (USA).

### Cell Culture

Hela and Hep2 cells were purchased from the Cell Bank of the Chinese Academy of Sciences (Shanghai, China). The cells were cultured in DMEM medium (Hyclone, Thermo Fisher Scientific, USA) and 10% fetal bovine serum (FBS, Millipore, USA). The cells were grown at 37 °C in an incubator with a 5% CO_2_ humidified atmosphere.

### Preparation of Silica Particles

In a typical synthesis, 0.55 g CTAB was completely dissolved in 25.0 ml of deionized water under stirring, and 3 g PAA (25 wt % solution) was added under vigorous stirring at room temperature to obtain a clear solution. Further, 2.0 g ammonia (25%) was added to the above solution under vigorous stirring. Immediately, the solution formed a milky suspension due to the formation of PAA/C16TA complexes. After further stirring for 20 min, 2.08 g tetramethyldisiloxane (TEOS) was added to the above solution and stirred for another 15 min. The mixture was transferred into an autoclave, which was put in an oven of 80 °C for 48 h. The white final product was centrifuged, washed with deionized water, and dried at 60 °C. The PAA and part of surfactants were removed by acid extraction and the detailed procedure was as followed: 1.0 gas-synthesized sample was stirred in a mixture solution of 100 ml acetonitrile and 10.42 g 36–38 wt% HCl for 24 h at room temperature. The product was filtrated, washed with deionized water and dried at 50 °C. The amino-functionalized mesoporous silica was obtained via a reaction between dried extracted mesoporous silica with APMS in toluene. Carboxyl-functionalized mesoporous silica was obtained via further reaction between dried amino-functionalized mesoporous silica with malice anhydride in toluene.

### Preparation of RGD-hylin a1-HMS

In order to link RGD-hylin a1 peptides with HMS-COOH, first 1 mg/ml RGD-hylin a1 peptides and 1 mg/ml HMS-COOH were mixed and gently shaken at room temperature (25 °C) for 48 h. The pH value of the mixture was adjusted to 10 using 0.5 M NaOH. The free RGD-hylin a1 peptides and RGD-hylin a1-HMS were separated by centrifugation at 6000 rpm for 10 min at 4 °C. The precipitation was washed by 10 mM PBS and then re-centrifuged. The process was repeated twice. Finally the RGD-hylin a1-HMS was dispersed in PBS (10 mM, pH 7.4) and stored at 4 °C.

### Characterization of HMS and RGD-hylin a1-HMS

The prepared HMS-COOH was observed by the transmission electron microscopy (H7650, Hitachi, Japan), the samples were scattered in pure water, dripped in copper mesh, filter papers were used to absorb excess moisture and dried for observation. The isotherms of nitrogen were measured using N_2_ adsorption/desorption system (Quantachrome Auto-sorb-1-C, America). Adsorption branches of N_2_ adsorption/desorption isotherms calculated HMS-COOH and RGD-hylin a1-HMS surface area by BET method, and desorption average pore diameter based on the BJH model. The content of RGD-hylin a1-HMS in aqueous solution was analyzed by HPLC (Shimadzu LC-10AT, SPD-10A, Japan) on a C18 column(Vydac C18, 218TP54, 4.6 × 250mm, Grace, America), equilibrated with solvent A (0.05% TFA) for 5 min, then elution was performed, at 1.0 ml/min, with a 0–60% linear gradient of solvent B (acetonitrile containing 0.05% TFA) for 20 min, 60%-100% of solvent B in 5 min and finally, washed with 100% solvent B for 5 min. The absorbance was measured at 220 nm.

### Cell Apoptosis Assays

Annexin V FITC Apoptosis Detection Kit purchased from BD (USA) was used to detect the cell apoptosis rates induced by the antimicrobial peptides. Hela and Hep2 cells were seeded in 12-well plates and incubated as 10^5^ cells per well. Various concentrations of antibacterial peptides were added to the wells every 24 h and incubated with cells at 37 °C in an incubator with a 5% CO_2_ humidified atmosphere. The cells were washed twice with cold PBS and then suspended in 1× Binding Buffer at a concentration of 1 × 10^6^ cells/ml after which 100 μl solution (1 × 10^5^ cells/ml) was transferred to a 5 ml culture tube, and 5 μl of FITC Annexin V and 5 μl PI added. The solution was gently vortexed and incubated for 15 min at room temperature (25 °C) in the dark. Then 400 μl of 1× Binding Buffer was added to each tube, and analyzed by flow cytometry within 1 hour. The dual fluorescent signals of the cells were analyzed using a micro capillary flow cytometer (BD, USA). Besides, the pH of cell culture medium was adjusted by acid and alkali solutions in the pH-dependent experiment.

### Detection of Mitochondrial Membrane Potential Changes

Flow Cytometry Mitochondrial Membrane Potential Detection Kit was purchased from BD (USA) and used to detect the mitochondrial membrane potential changes. The preparation of cell samples was the same as that in the cell apoptosis assays. The 1 ml cell solution (1 × 10^6^ cells/ml) was transferred to a 15 ml culture tube, centrifuged at 800 rpm for 5 min, after discarding the supernatant, 0.5 ml of JC-1 working solution was added. The solution was gently vortexed and incubated for 15 min at room temperature (25 °C) in the dark. The precipitate was washed at 800 rpm for 5 min by 1 ml 1× Assay Buffer and then re-centrifuged. The process was repeated twice, and then the precipitate re-suspended by adding 0.5 ml 1× Assay Buffer. Finally, the dual fluorescent signals of the 0.5 ml solution were analyzed using a micro capillary flow cytometer (BD, USA).

### Hemolysis Assays

Human blood was obtained from the First People’s Hospital of Shangqiu. Blood samples were washed several times by 10 mM PBS and centrifuged until a clear supernatant was obtained, 2% blood samples were then prepared. Various concentrations of antibacterial peptides were incubated with 500 μL of 2% blood samples at 37 °C for 1 h. The absorbance of the supernatants from each group of blood samples was measured using UV-vis spectrophotometer (UNIC-2100, Shanghai, China) at 540 nm. 2% blood samples that had been treated with 1% Triton were used as positive controls, and the release rate of hemoglobin for this group was set at 100%. The release rate of antibacterial peptides measured by hemolysis assays was performed as follows: first released antibacterial peptide samples at relevant pHs and time periods, then incubated 50 μl of the released antibacterial peptide samples with 950 μl of 2% blood samples (pH = 7) at 37 °C for 1 h (The final pH of the solution was still 7).

### Antitumor Studies *in vivo*

All animal studies had been approved by the Jiangsu Provincial Animal Care and Use Committee and the experimental guidelines of the Animal Experimentation Ethics Committee of Nanjing Agricultural University. Balb/c-nu mice (half female and half male, 6 weeks old) were implanted subcutaneously with Hep2 cells in the left flank. When the tumor volume grew to 100 mm^3^, the mice were randomly separated into 4 groups. Each group contained 5 mice. The 4 groups of tumor-bearing mice were administered with HMS-COOH (10 mg/kg), RGD-Hylin a1 (10 mg/kg), HMS-hylin a1-RGD (10 mg/kg) or PBS respectively via tail vein injection for 6 days. The body weight and tumor size of each mouse were measured using an electronic balance and calipers on the 1st (the first day of injection), 3rd, 5th, 7th, 9th days. The tumor volumes were calculated according to the following formula: V = 0.5 × L(length) × W(width) × H(height)^40^. On the 9th day, all of the mice were killed and the tumors were dissected, photographed, and weighed. The inhibition rates of tumor growth were calculated according to the following formula: inhibition (%) = (C−T)/C × 100, where C was the average volume of the tumor in the control group (PBS) and T was the average volume of the tumor in the treated group.

### Hemanalysis and Biochemical Analysis

Blood samples were collected before the mice were killed. The blood cell and biochemical analysis were performed using hematology analyzer (SYSMEX, XE-5000, Japan) and a biochemical analyzer (Hitachi Model 7600 Series Automatic Analyzer, Japan), respectively.

### Histopathologic Analysis of Normal Tissues

The hearts, livers, spleens, lungs, and kidneys were extracted from the tumor-bearing mice and fixed in 4% paraformaldehyde solution. The organs were embedded in paraffin, sectioned, and stained with hematoxylin and eosin (H&E). The images were obtained using Zeiss laser microscope (Zeiss, Germany).

### Statistical Analysis

SPSS 17.0 statistical software was used for data analysis in the studies and all the experiments were done in triplicates. The results are given as mean ± SD. Comparisons between two groups were done using Student’s t test analysis. Multiple comparisons were done with one-way analysis of variance followed by Fisher’s least significant difference analysis. Significant differences between or among groups were indicated by * for p < 0.05, ** for p < 0.01, and *** for p < 0.001.

### Ethical conduct of research

The authors state that they have obtained the Animal Experiment Committee of Nanjing Agricultural University approval and have followed the principles outlined in the Declaration of Helsinki for all human or animal experimental investigations.

## Electronic supplementary material

Supplementary Information
