# Supplementary material for: A pH-dependent Antibacterial Peptide Release Nano-system Blocks Tumor Growth in vivo without Toxicity
Source: Sci Rep. 2017 Sep 11;7:11242. doi: 10.1038/s41598-017-11687-y (PMC5593885; doi:10.1038/s41598-017-11687-y)
Supplement: Supplementary file 1 — Supplementary Information [file 41598_2017_11687_MOESM1_ESM.pdf]

A pH-dependent Antibacterial Peptide Release Nano-system Blocks Tumor Growth *in vivo* without Toxicity

Jing Cao[1, 2], Yan Zhang[1], Yanke Shan[1], Jingui Wang[3], Fei Liu[1*], Hongrui Liu[1], Gang Xing[1], Jing Lei[1], Jiyong Zhou[1]

[1]Engineering Laboratory of Animal Immunity of Jiangsu Province, Institute of Immunology and College of Veterinary Medicine, Nanjing Agricultural University, Nanjing, Jiangsu 210095, P.R. China.
[2]College of biology and Food Science, Shangqiu Normal University, Shangqiu, Henan 476000, P.R. China
[3]Shandong Provincial Key Laboratory of Fine Chemicals, School of Chemistry and Pharmaceutical Engineering, Qilu University of Technology, Jinan, P.R. China.

*Send correspondence to:

Fei Liu, Engineering Laboratory of Animal Immunity of Jiangsu Province, Institute of Immunology and College of Veterinary Medicine, Nanjing Agricultural University, Nanjing, Jiangsu 210095, P.R. China.

Email: feiliu24@njau.edu.cn

Supporting Information

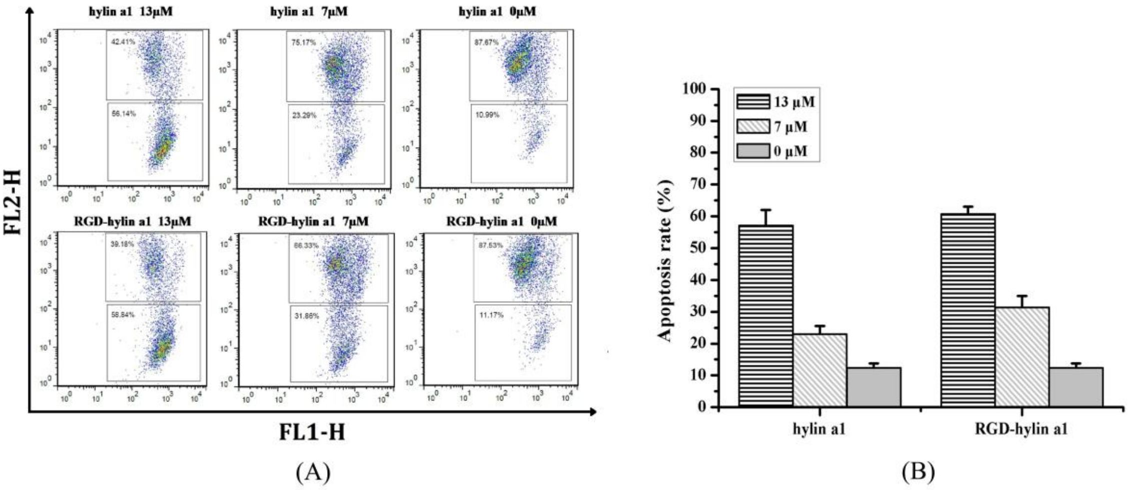

Figure S1. Mitochondrial membrane potential assays with hylin a1 or RGD-hylin a1 treatment. (A) and (B) Hela cells were treated with hylin a1 and RGD-hylin a1 for 24 h, stained with JC-1, and analyzed by FACS. Results were presented as percentage of apoptosis.

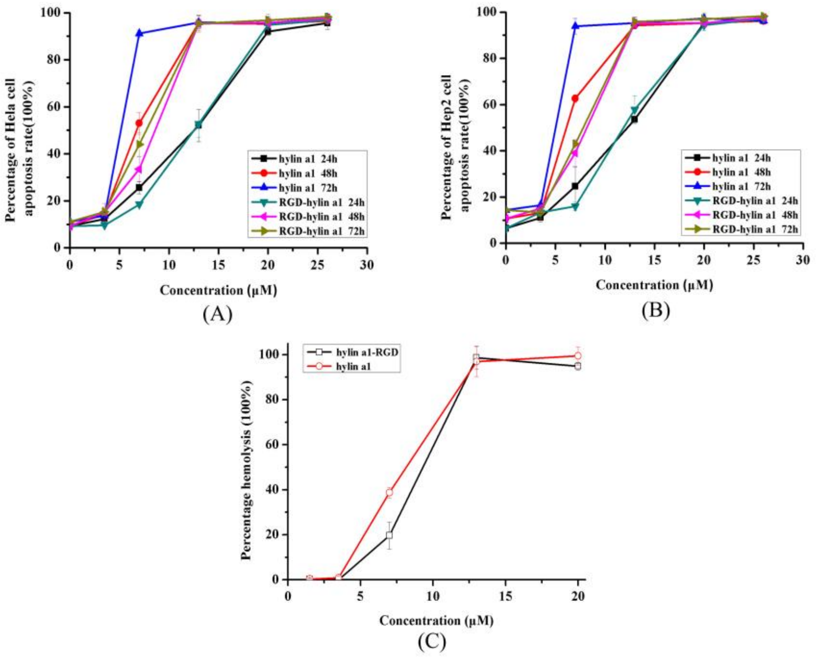

Figure S2. Comparison of cytotoxicity assays and hemolysis assays of hylin a1 and RGD-hylin a1. All data was presented as the means ± SD, n=3. (A) Apoptosis assays evaluating the cytotoxicity of hylin a1 and RGD-hylin a1 in Hela cells. (B) Apoptosis assays evaluating the cytotoxicity of hylin a1 and RGD-hylin a1 in Hep2 cells. (C) Hemolysis assays for hylin a1 and RGD-hylin a1 in RBC.

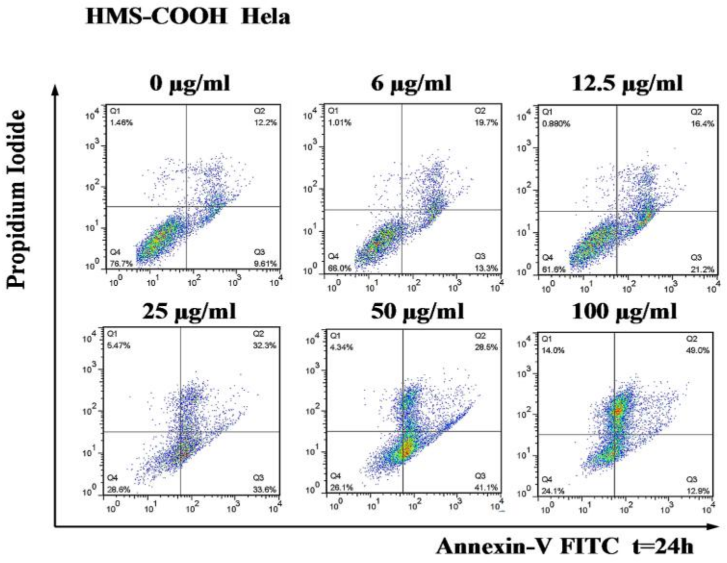

Figure S3. Cytotoxicity assays in Hela cells. Hela cells were treated with HMS-COOH for 24 h, stained with annexin V and PI.

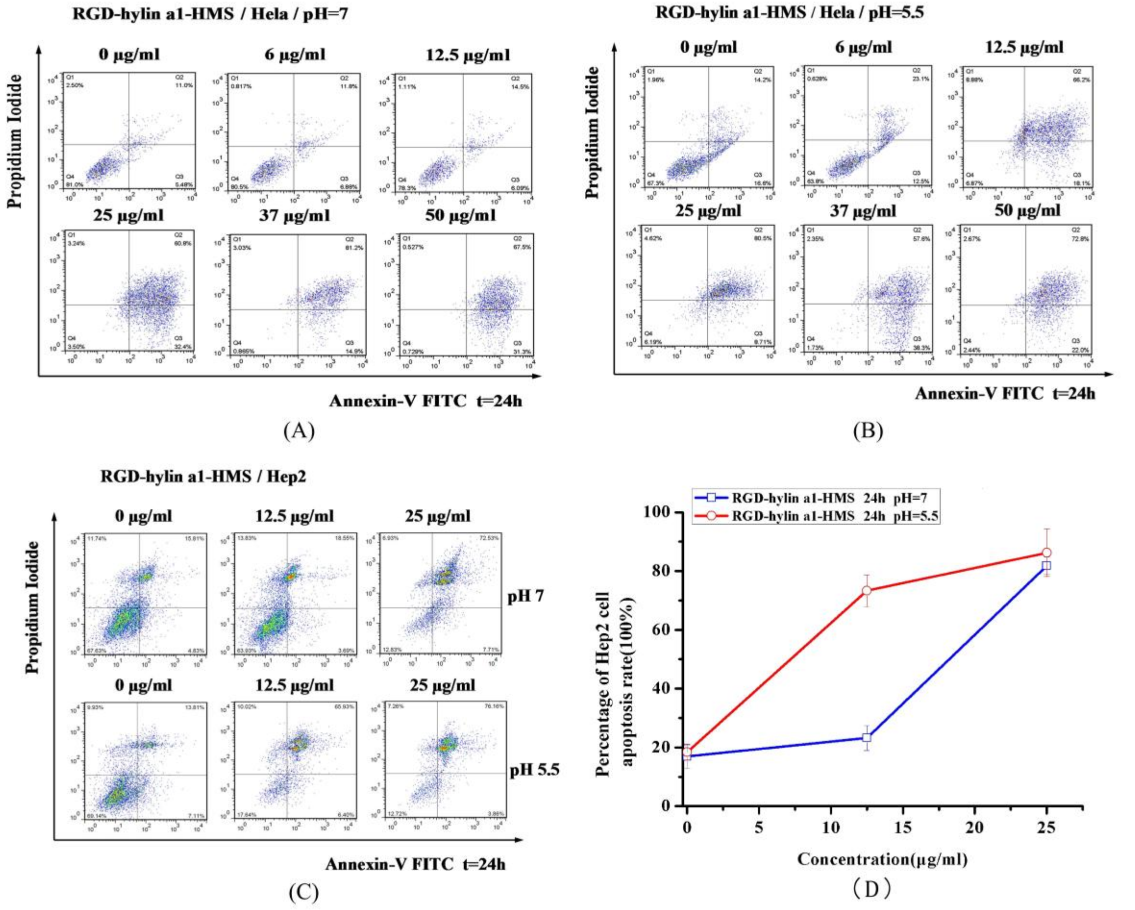

Figure S4. Cytotoxicity assays of RGD-hylin a1-HMS in Hela and Hep2 cells. (A) Hela cells were treated at pH = 7 for 24h. (B) Hela cells were treated at pH = 5.5 for 24h. (C) and (D) Hep2 cells were treated for 24h at pH = 7 or pH = 5.5.

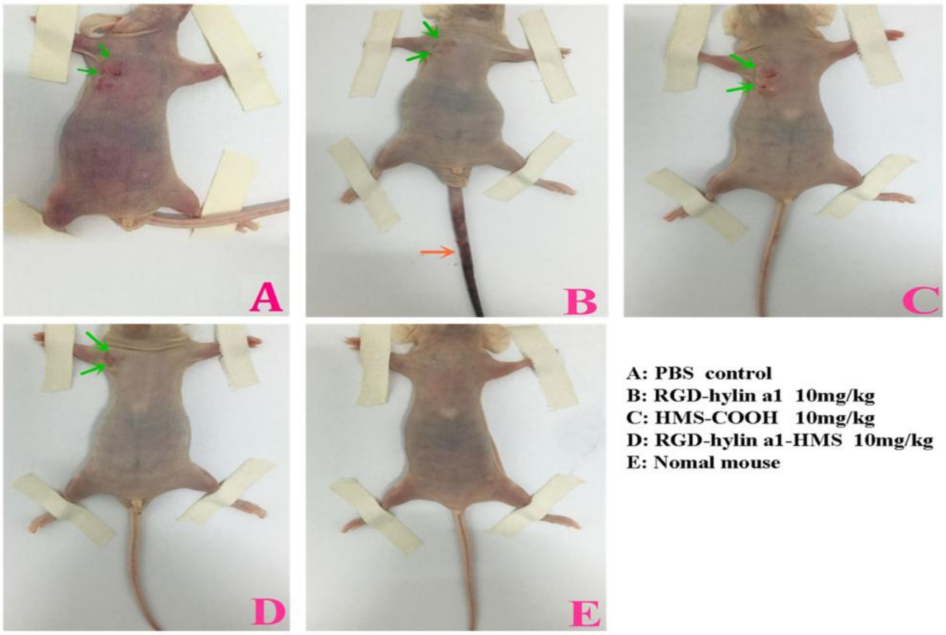

Figure S5. Photographs of tumor bearing nude mice. Green arrows indicate where the solid tumors are. Red arrow indicates the the tail of the nude mice festered seriously with just RGD-hylin a1 treatment.

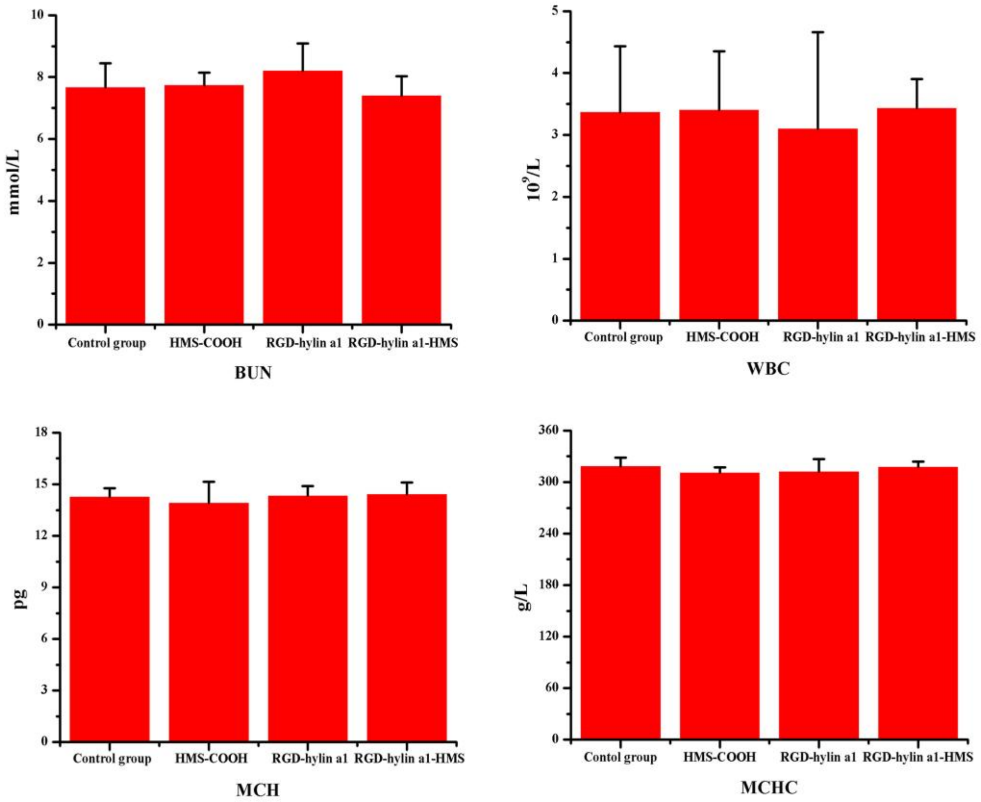

Figure S6. Evaluation of the side effects of RGD-hylin a1-HMS *in vivo*. All data was presented as the means ±SD, n = 3, *p < 0.05, **p < 0.01. BUN: Blood urea nitrogen, WBC: Blood hemanalysis of white bood cells, MCH: mean cell hemoglobin, and MCHC: mean corpuscular hemoglobin concentration.
